# Machine learning-based anomaly detection of groundwater microdynamics: case study of Chengdu, China

**DOI:** 10.1038/s41598-023-38447-5

**Published:** 2023-09-07

**Authors:** Haoxin Shi, Jian Guo, Yuandong Deng, Zixuan Qin

**Affiliations:** 1 State Key Laboratory of Geohazard Prevention and Geoenviromment Protection, Chengdu University of Technology, Chengdu, 610059 China; 2 College of Construction Engineering, Jilin University, Changchun, 130026 China; 3 College of New Energy and Environment, Jilin University, Changchun, 130026 China

**Keywords:** Environmental sciences, Hydrology

## Abstract

Detection of subsurface hydrodynamic anomalies plays a significant role in groundwater resource management and environmental monitoring. In this paper, based on data from the groundwater level, atmospheric pressure, and precipitation in the Chengdu area of China, a method for detecting outliers considering the factors affecting groundwater levels is proposed. By analyzing the factors affecting groundwater levels in the monitoring site and eliminating them, simplified groundwater data is obtained. Applying sl-Pauta (self-learning-based Pauta), iForest (Isolated Forest), OCSVM (One-Class SVM), and KNN to synthetic data with known outliers, testing and evaluating the effectiveness of 4 technologies. Finally, the four methods are applied to the detection of outliers in simplified groundwater levels. The results show that in the detection of outliers in synthesized data, the OCSVM method has the best detection performance, with a precision rate of 88.89%, a recall rate of 91.43%, an F1 score of 90.14%, and an AUC value of 95.66%. In the detection of outliers in simplified groundwater levels, a qualitative analysis of the displacement data within the field of view indicates that the outlier detection performance of iForest and OCSVM is better than that of KNN. The proposed method for considering the factors affecting groundwater levels can improve the efficiency and accuracy of detecting outliers in groundwater level data.

## Introduction

Groundwater is an essential and important water resource for human society. Unreasonable extraction of groundwater can easily lead to a decline in groundwater levels, causing soil erosion and the loss of wetlands, which has a severe impact on the resource environment and ecological sustainability^1, 2^. At the same time, the rapid development of economic activities has led to an increasing degree of environmental pollution of groundwater^3^. In order to strengthen groundwater management, prevent overextraction and pollution of groundwater, ensure the quality and sustainable use of groundwater, relevant departments in various countries have set up a large number of monitoring wells throughout the country^4–7^. These monitoring wells record the rich information changes of groundwater levels, temperatures, water chemistry ions etc. in real time, providing decision-making bases for reasonable utilization and scientific management of groundwater. However, how to timely and accurately analyze the information in groundwater is the key factor that affects decisions.

During the entire monitoring process, failures of sensors and external factors may cause abnormal monitoring data. The existence of abnormal values may cause significant uncertainty in interpretation results. On the one hand, abnormal values caused by sensors need to be rejected as misleading data^8^. On the other hand, abnormal values caused by external factors may show sudden increases or decreases in groundwater levels (temperatures) or sudden changes in water chemistry characteristics^9, 10^. Therefore, before conducting any further analysis, it is essential to perform abnormal data detection on the monitoring data^11^.

Existing anomaly detection methods can be classified into three main categories: statistical methods^12^, regression techniques^13^, and machine learning methods^14^, and have been widely studied and applied in industrial fault checking, network intrusion detection, medical diagnosis, fraud detection, time series anomaly detection and various other fields^15–19^. In the face of different application fields and different data types, the same outlier detection method cannot all achieve good detection results^20^, so it is necessary to choose the appropriate Outlier detection techniques according to the type of data being studied. Groundwater monitoring data, as a type of time series data, has the following difficulties in outlier detection: first of all, when detecting outliers in time series, it is necessary to ensure the timeliness of data information, that is, the detection mechanism must have the ability to continuously learn new data, and perform real-time analysis to detect outliers; However, these laws usually have a certain time span, which inevitably increases the difficulty of outlier detection. In addition, the existence of outliers in time series is usually a small probability event, and the small sample size of outliers also leads to the difficulty of outlier detection. Therefore, some traditional anomaly detection methods that do not consider the time factor do not apply to the detection of time series outliers.

Since the 1990s, several scholars have explored and studied the problem of outlier detection in time series^20–27^. Among them, statistical-based time series detection methods are based on massive data, and the model created by existing or assumed data knowledge is used as a normal profile, and the data points that do not fit in the model are marked as outliers, and the main detection methods are Z-value method^28^, Pauta criterion method^29^, quadratic method^30^, and point distribution ESD test^31^. The regression-based detection method takes time as the independent variable and the corresponding time series data as the dependent variable, establishes a time series regression model, uses the predicted data of the model and the actual data to compare the difference, and then analyzes the deviation degree of the residual to determine the Judging the abnormal data position of the time series^32^, and the main detection methods are regression model (AR), moving average model (MA), autoregressive moving average model (ARMA), and autoregressive summation moving average model (ARIMA). After entering the 21st reality, with the popularization and development of computer technology, the outlier detection methods based on machine learning have become more and more popular among researchers, and Fei et al.^33, 34^ proposed the isolated forest (iForest) algorithm based on the classification idea, which was improved by scholars and widely used in data anomaly detection and achieved better prediction results^35–38^; Support Vector Machines (SVM) algorithm is also based on the classification idea and has been used by a wide range of scholars to analyze time series problems^39–42^; K-nearest neighbours (KNN) algorithm is based on the idea of distance, based on distance metrics (e.g. Eucledian or Manhattan distance) to calculate the average distance to the k-nearest neighbors, and the points with the maximum average distance are marked as outliers^43, 44^. The above outlier detection algorithm identifies abnormal data points of water consumption in groundwater^45^, water temperature abnormal points^46^ and water quality abnormal monitoring^47, 48^ address some outlier detection problems in groundwater and monitoring wells.

However, less research has been conducted on the detection of anomalous data in high-frequency high-precision groundwater level real-time dynamics. Azimi et al.^49^ used the Monte Carlo method and importance sampling to determine the long-term variation of groundwater level, and then determined the groundwater anomaly pattern based on the SVM, iForest algorithm and robust covariance algorithm. Find the best outliers, locate unusual aquifers and explore connections between aquifers. However, the inhomogeneity of the research sites may lead to errors in the analysis results, and in order to limit the long-term and short-term non-stationary processes of high-frequency data, the hyperparameter optimization of the algorithm has not been fully studied.

In 2020, Liu et al.^50^ attempted to use one-class support vector machine (OCSVM) technology to perform real-time anomaly monitoring on real groundwater data in Colorado, and through numerical simulation of pollutant migration in porous media. The obtained synthetic data was used to verify the model experimentally, and a good detection success rate was obtained, but they only examined the OCSVM technique and did not validate it against other anomaly detection techniques. From this, it can be found that the current research mainly focuses on the direction of data-driven detection alone such as optimization algorithm and combination algorithm, without considering the judgment of outlier detection based on the essential characteristics of groundwater.

The groundwater level data collected through actual monitoring is not labeled, and there is no information about the type and number of abnormal values. Therefore, it is difficult to directly evaluate the performance of different abnormal value detection methods. Therefore, the main purpose of this article is to propose an abnormal value detection method for groundwater microdynamic real-time data that takes into account the data structure, in order to improve the detection rate of abnormal groundwater levels. Firstly, This study first detects known abnormal values in a time series, and then evaluates the accuracy of the four methods using indicators such as accuracy, precision, and ROC curve. Then, the influencing factors of groundwater levels in the monitoring site in Chengdu, China are analyzed, and the fixed impact features are removed through inverse convolution and filtering to simplify the data structure. Finally, abnormal value detection is performed on the simplified data. The results are compared with the soil displacement data of the site to investigate the performance of the detection method that takes into account the data structure.

## Materials and method

### Overall framework

**Figure 1:**
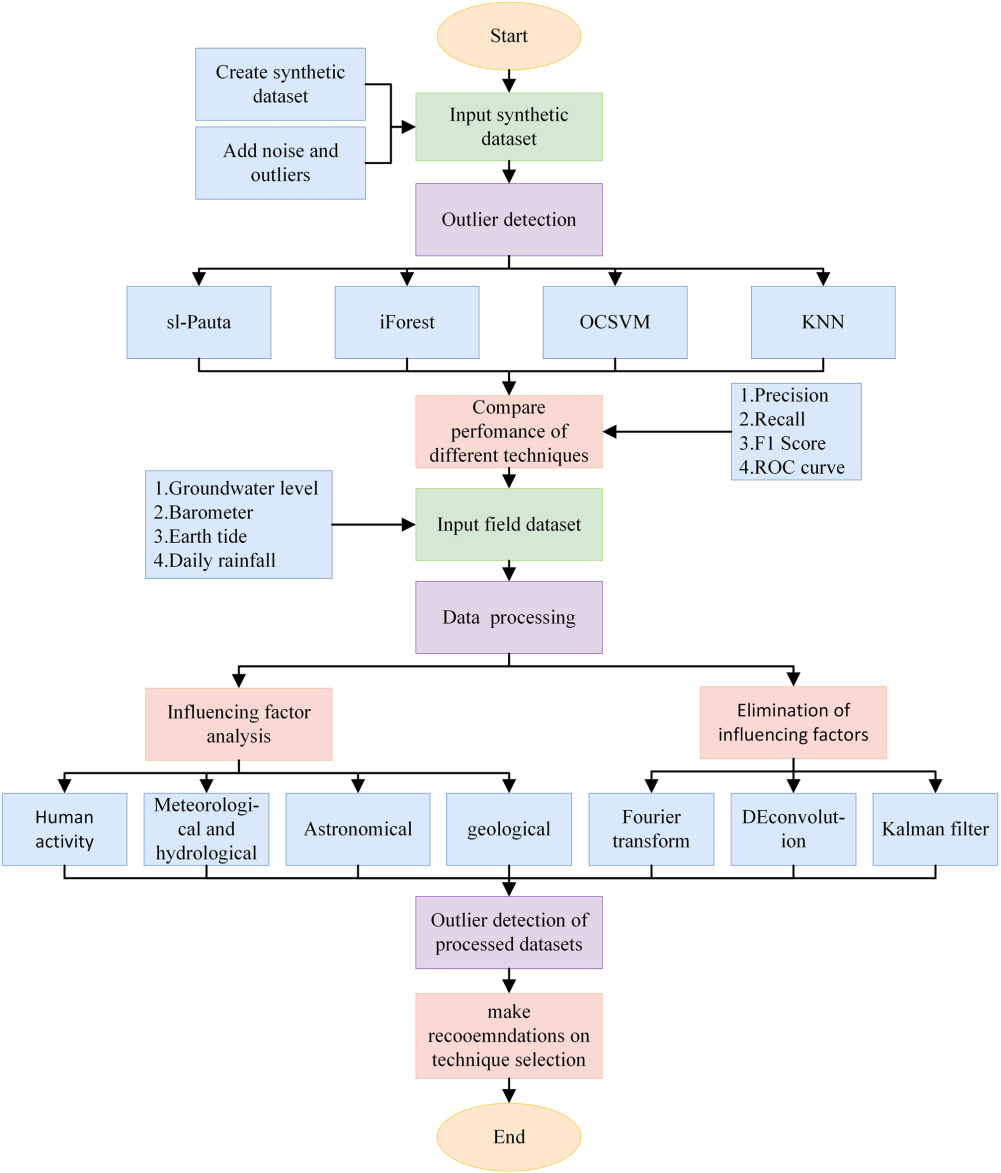
Machine learning framework for groundwater microdynamics anomaly detection.

As shown in Fig. 1, a detection model was established through the following three steps. Firstly, a dataset containing noise and abnormal values was created and analyzed using four algorithms: sl-Pauta, iForest, OCSVM, and KNN. The accuracy of the detection algorithm was determined through performance evaluation. Secondly, real-time monitoring data was collected and factors affecting the scene were analyzed using Fourier transformation and spectral analysis to determine the frequency of relevant factors. The relevant factors were then removed using inverse convolution and filtering methods to obtain simplified water level data. Finally, the four algorithms were used to detect abnormal values in the processed data, and the performance of different techniques was qualitatively compared using displacement data using professional knowledge.

### Model algorithms

#### Self-learning-based Pauta criterion method

**Figure 2:**
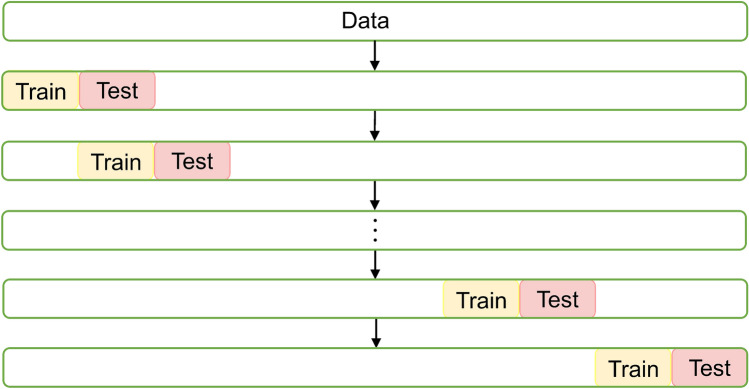
Flow chart of sl-Pauta criterion to detect outliers.

The Pauta criterion is a statistical method for detecting outliers in sample data. When applying it to the detection of abnormal water levels in underground mines, the accuracy of using a certain section of water level data to detect overall data outliers is limited. Therefore, based on the basic principles of the Pauta criterion, an automatic learning function is added by automatically moving down one position every time a water level data point is detected. The algorithm is shown in Fig. 2, where the Train is water level data used to calculate the mean $\overline{x }$ and standard deviation $\sigma$, and the Test points to the water level data that needs to be detected. Every time the water level of a point is detected, the training segment and testing segment move forward by one position as a whole, allowing the Pauta criterion to achieve automatic learning, which includes the function of adding the latest running results to the mean and standard deviation in real time, resulting in more accurate detection results. The specific process of the algorithm can be found in Appendix 1.1.

1 $$\sigma =\sqrt{\frac{\sum_{i=1}^{n}{x}_{i}-\overline{x} }{n-1}}$$

#### Isolated forest algorithm

The Isolation Forest (iForest) algorithm is an unsupervised anomaly detection method based on random binary trees, which is suitable for continuous data. The algorithm defines an anomaly as "easily isolated anomalous values", which are points that are sparsely distributed and far away from high-density populations in the feature space. In the feature space, areas with sparse distribution indicate that the probability of occurring the event is very low, so it is inferred that data points distributed in sparse regions are anomalous values. The iForest algorithm consists of K trees, each tree is a binary tree. The algorithm randomly selects a subset of samples as the root node, then randomly selects a feature as the cutting point to generate a binary tree. It continuously constructs new leaf nodes until the maximum depth of the binary tree or the final leaf node has only one data point, which generates the completed isolated tree. During the training process, each iTree is randomly selected and independently generated. The more iTrees are used, the more stable the algorithm is. The evaluation formula for anomalous values is as follows:

2 $$s\left(x,\psi \right)={2}^{-\frac{E(h(x))}{c(\psi )}}$$

In the formula, $c(\psi )$ is the average path length of samples with a given number of $\psi$, which is used to standardize the path length $h(x)$ of sample $x$, $h(x)$ is the height of $x$ in each tree. If $s$ is closer to 1, the possibility of it being an anomalous point is higher. If $s$ is much smaller than 0.5, it is definitely not an anomalous point. If $s$ is around 0.5, there is no possibility of there being anomalous points in the dataset.

#### One-Class SVM

One-Class SVM was proposed by Bernhard Schölkopf et al. in 2000, and the method is often used to monitor abnormal behavior in data^51^. The basic idea is to compute a hypersphere with the smallest radius in a sample and to include all samples inside this hypersphere. When this hypersphere is used to classify the dataset, the samples that fall inside the hypersphere are the first class (normal values) and the samples that fall outside the hypersphere are the second class (abnormal values). In this method, the kernel and scalar parameters need to be selected to determine the decision boundary and the parameter optimization problem. OCSVM separates a region of hyperspace with more data points close to each other from another data point with less density by computing a decision boundary, and treats these points as anomalies.

The function of the optimization problem is shown in Eqs. (3–5):

3 $${min}_{w,{\varepsilon }_{i},\rho }=\frac{1}{2}{\Vert w\Vert }^{2}+\frac{1}{vn}{\sum }_{i=1}^{n}({\varepsilon }_{i}-\rho )$$

4 $$\left(w,\varnothing \left({x}_{i}\right)\right)\ge \rho -{\varepsilon }_{i} \mathrm{for }i=1,\dots ,n$$

5 $${\varepsilon }_{i}\ge 0 \mathrm{for }i=1,\dots ,n$$

The determination function of the decision boundary is shown in Eq. (6):

6 $$f\left(x\right)=sgn(\left(w,\varnothing \left({x}_{i}\right)-\rho \right)\Rightarrow f\left(x\right)=sgn\left({\sum }_{i=i}^{n}{\alpha }_{i}K\left({x}_{i},x\right)-\rho \right)$$

#### KNN

The KNN algorithm is based on the distance for data segmentation and outlier detection^52^. The basic logic is to calculate the average distance between each sample point and its nearest K samples in turn and then use the calculated distance to compare it with a threshold, and if it is greater than the threshold, it is considered an outlier. Use the Euclidean distance ${d}_{xy}$ formula to calculate the distance between the current point and each point in the data set., sort the points in increasing order of distance, select the k points with the smallest distance from the current point; calculate the distance between the current point and its k neighbors, and take one of the mean, median, or maximum value as the outlier.

7 $${d}_{xy}=\sqrt{\sum_{k=1}^{n}{({x}_{k}-{y}_{k})}^{2}}$$

### Algorithm evaluation

The algorithmic evaluation of outlier detection is considered a dichotomous problem, where the labels can be divided into 0 and 1, and a mixture matrix is constructed (Table 1), which acts as the true case and is listed as the detection result.

**Table 1:** Confusion matrix.

Confusion matrix	Predict	
	Positive 1	Negative 0
Real		
True 1	TP	FN
False 0	FP	TN

TP: true value is 1, predicted value is 1, prediction is correct; FP: true value is 0, predicted value is 1, prediction is wrong; FN: true value is 1, predicted value is 0, prediction is wrong; TN: true value is 0, the predicted value is also 0, the prediction is correct.

**Figure 3:**
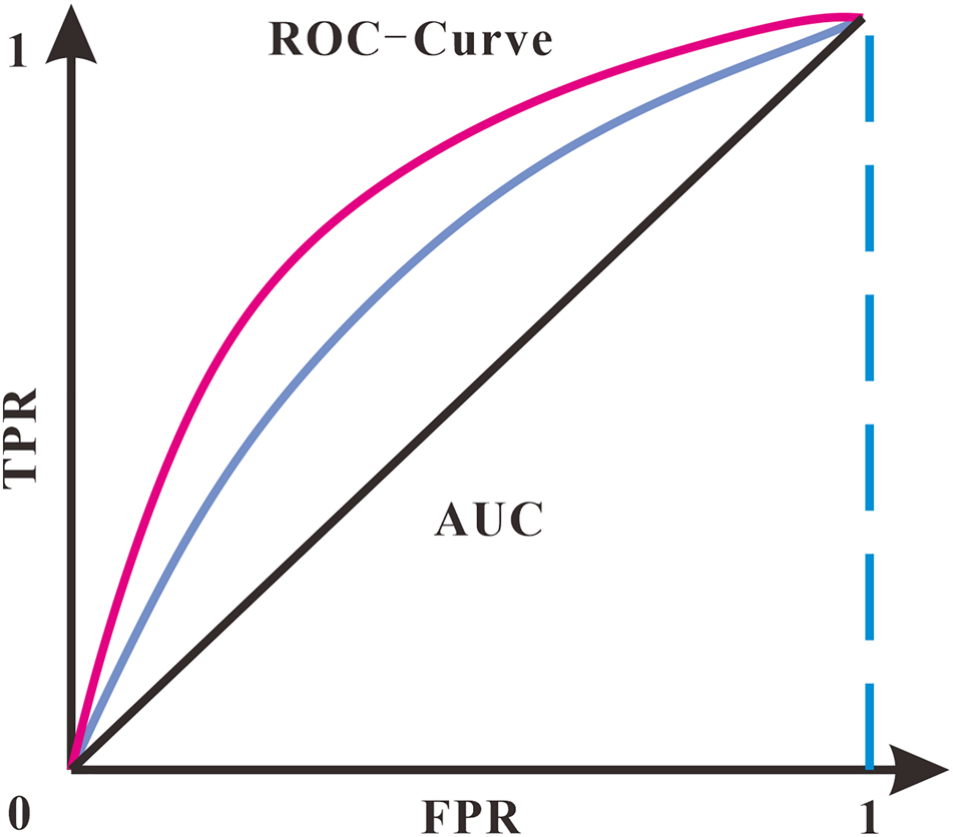
ROC curve.

The evaluation of the results can be judged by precision rate, recall rate, F1 score, and ROC curve. Precision is the probability of the actual positive samples among all the predicted positive samples (the proportion of samples with a prediction of 1 and a true label of 1), which is the prediction result, as shown in Eq. (6). Recall is the probability of the actual positive samples being predicted as positive samples (Eq. 7). The false positive rate (FPR) represents the probability of misclassifying a negative case as a positive case (Eq. 8). The F1 score is equalized to the evaluation metric as a summed average of the precision rate and recall rate (Eq. 9). The ROC curve consists of a series of points (Fig. 3), and each point on the curve corresponds to the classification result when the classifier takes a certain threshold, with the true rate (TPR) as the vertical coordinate and the false positive rate (FPR) as the horizontal coordinates, the points where the threshold value changes move in the direction of the arrow in the graph. In the ROC graph, the closer the curve is to the point (0,1), the better the classification effect, that is, the steeper the curve, the better the evaluation result. Since the ROC curve lacks numerical representation, the area enclosed by the ROC curve and the x-axis (AUC, Area Under Curve) is usually used to evaluate the merit of the result, that is, the closer the AUC is to 1, the better the evaluation result:

8 $$\mathrm{Precicion}=\frac{TP}{TP+FP}$$

9 $$\mathrm{Recall}=\frac{TP}{TP+FN}$$

10 $$\mathrm{FPR}=\frac{FP}{FP+TN}$$

11 $$\mathrm{F}1=\frac{2*Precision*Recall}{Precision+RecallP}$$

### Data source

**Figure 4:**
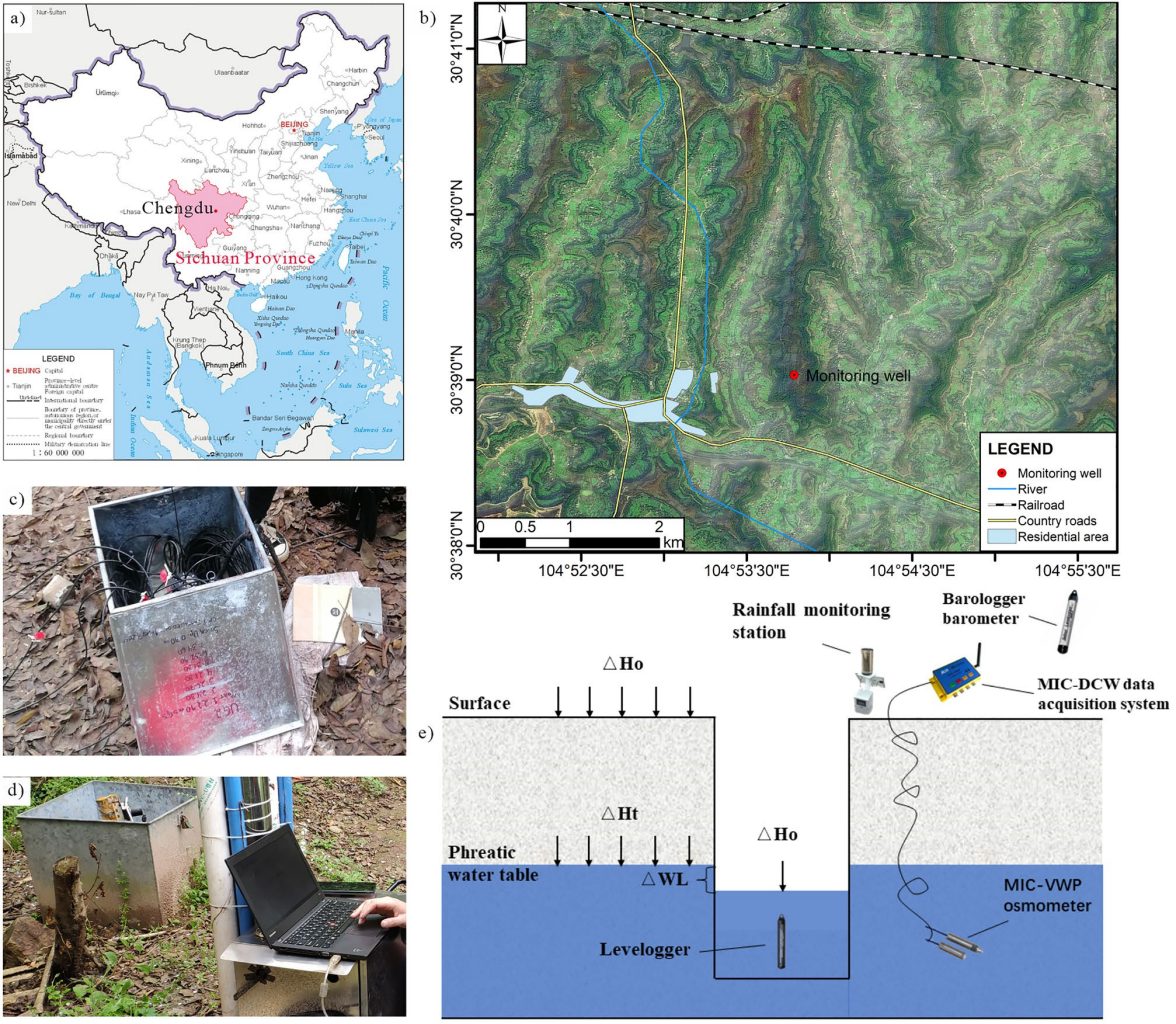
Study area and monitoring equipment ((**a**) is the geographic location of the study area; (**b**) is an overview of the study area site; (**c**) is a photo of the monitoring well site; (**d**) is regular on-site maintenance of equipment and review of data; (**e**) is a sketch of the monitoring well layout).

The study area is located on the Kualiangzi landslide in Chengdu, Sichuan Province, China. The team has deployed a series of monitoring equipment in the area, including rain gauges, water level gauges, pore water pressure gauges, barometers, and displacement gauges in the area (Fig. 4) to monitor rainfall, Monitoring of groundwater, air pressure, displacement, etc.

**Figure 5:**
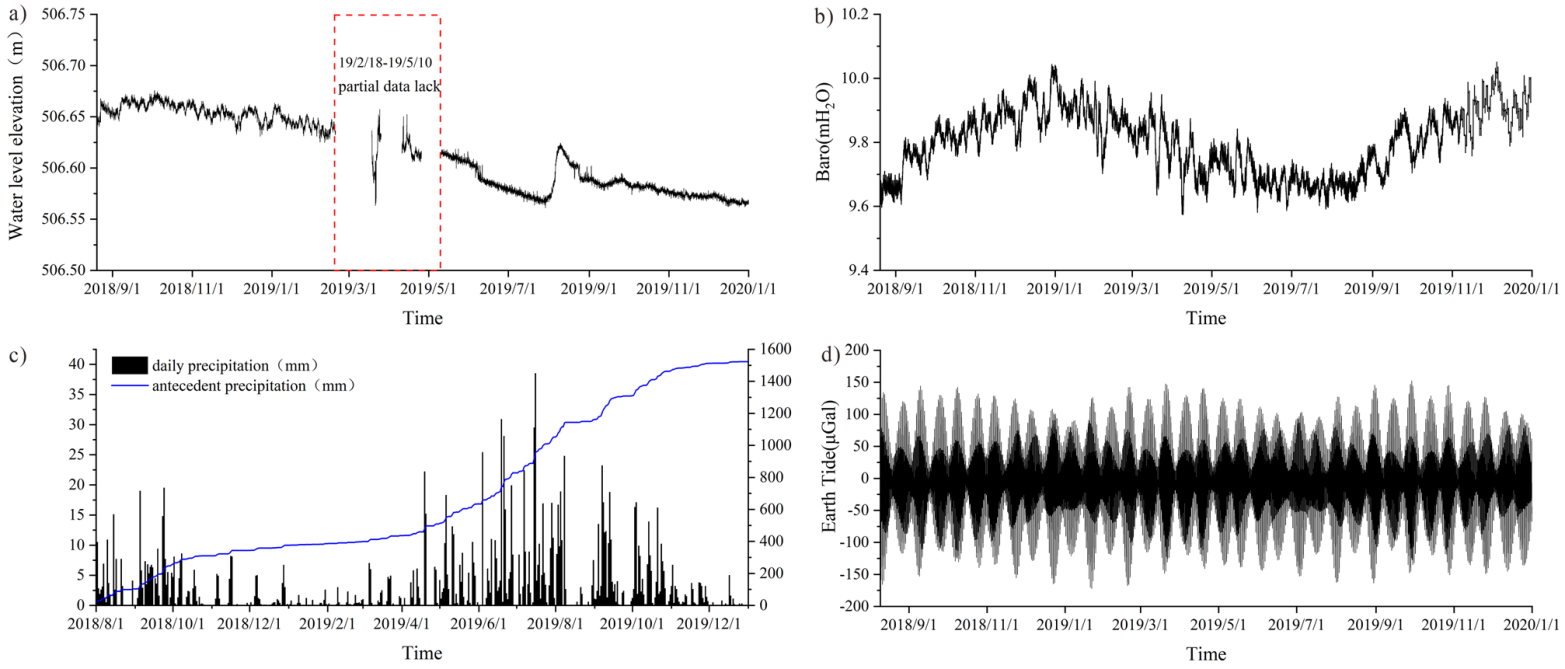
Basic data of the study area ((**a**) is water level elevation data; (**b**) is barometric data; (**c**) is antecedent precipitation data; (**d**) is earth tide data)).

Groundwater data were obtained by RST vibrating wire piezometer, the vibrating wire transducer output frequency signal, this frequency is proportional to the pressure applied to the vibrating wire piezometer and is not affected by wire impedance or contact resistance. These sensors can be installed in boreholes or driven into soft ground, the precision of the sensor is 1 mm, and the acquisition frequency is set to 1 time/h. A total of 10,813 well water level monitoring data were selected for research in two time periods from 0:00 on August 20, 2018 to 16:00 on February 18, 2019, and from 16:00 on May 10, 2019 to 23:00 on December 31, 2019. (part of the data from February 18, 19 to May 10, 19 was missing, so this time period was not studied), the well water level variation curve is shown in (Fig. 5a).

The barometric data are obtained from the Baro-Diver barometer, which is placed in the automatic data acquisition device next to the monitoring wells, and the atmospheric pressure in the study area is monitored in real-time at a frequency of 1 time/hour. Since the unit of atmospheric pressure and the unit of water level need to be unified when calculating the atmospheric pressure efficiency later, in order to put the atmospheric pressure and water level together, "mm water column" is used to represent the atmospheric pressure in this paper (Fig. 5b).

The precipitation data were collected by the tipping bucket type automated real-time precipitation monitoring point, which provides a real-time dynamic recording function for the frequency and intensity of precipitation in the area. The transmission interval of monitoring data is 1 time/h, and the precipitation data from August 1, 2018, to December 31, 2019, are finally obtained (Fig. 5c). According to the precipitation monitoring results, it can be visualized that the precipitation in the landslide area is mainly concentrated in the flood season from May to October, and the accumulated precipitation amounts to 1500 mm during the monitoring time.

The earth tide monitoring data were obtained from the OSG-066 superconducting gravimeter located in Lijiang at the Innovation Academy for Precision Measurement Science and Technology, Chinese Academy of Sciences (CAS), and further converted using Tsoft software^53^, the earth tide data from Lijiang were converted to data from the study area, and the time interval for obtaining the data was 1 h. The earth tide variation curve is shown in (Fig. 5d).

### Data processing

Kalman filtering method is used to eliminate the effect of precipitation in groundwater levels. Digital filtering is a method to highlight the effective frequency band waves by using the difference in spectral characteristics to suppress the interference waves in some frequency bands. The basic principle is to filter the irrelevant frequency bands by using the different frequencies of the desired information and the irrelevant information.

Rasmussen and Crawford in 1997 used the inverse convolution regression method to estimate the barometric response function using water level-barometric pressure observations and, through the response function, to calculate the corrected head^54^. The inverse convolution regression method can be used to eliminate both the barometric effect in water level and the effect of earth tide on water level, and it is ideal for use in unconfined aquifers. The air pressure response function using water level data and air pressure observation data:

12 $$\Delta \mathrm{W}\left(\mathrm{t}\right)=\mathrm{\alpha }\left(0\right)\Delta \mathrm{B}\left(\mathrm{t}\right)+\mathrm{\alpha }\left(1\right)\Delta \mathrm{B}\left(\mathrm{t}-1\right)+\mathrm{\alpha }\left(2\right)\Delta \mathrm{B}\left(\mathrm{t}-2\right)+\cdots +\mathrm{\alpha }\left(\mathrm{m}\right)\Delta \mathrm{B}\left(\mathrm{t}-\mathrm{m}\right)$$

that is:

13 $$\Delta \mathrm{W}\left(\mathrm{t}\right)=\sum_{i=0}^{m}\alpha \left(i\right)\Delta \mathrm{B}\left(\mathrm{t}-\mathrm{i}\right)$$

where $\Delta \mathrm{W}\left(\mathrm{t}\right)$, $\Delta \mathrm{B}\left(\mathrm{t}\right)$ are the values of the change in water level and atmospheric pressure at moment $\mathrm{t}$, $\Delta \mathrm{B}\left(\mathrm{t}-\mathrm{i}\right)$ is the value of the change in barometric pressure at moment $\mathrm{t}-1$, $\mathrm{\alpha }\left(\mathrm{i}\right)$ is the unit response coefficient at lag $i$, $m$ is the maximum lag time.

The unit response coefficient $\mathrm{\alpha }$ is found using least squares linear regression. once the value of $\mathrm{\alpha }(j)$ is found, the stepwise barometric response coefficient $\mathrm{A}\left(\mathrm{i}\right)$ is calculated by summing over the unit response coefficients:

14 $$\mathrm{A}(\mathrm{i})=\sum_{j=1}^{i}\alpha \left(j\right)$$

Using the response function, calculate the correction value for each observation:

15 $$\left[\begin{array}{c}{W}_{m}^{*}\\ {W}_{m+1}^{*}\\ {W}_{m+2}^{*}\\ \vdots \\ {W}_{n}^{*}\end{array}\right]=\left[\begin{array}{c}\begin{array}{c}{\Delta B}_{1}\\ {\Delta B}_{2}\\ {\Delta B}_{3}\end{array}\\ \vdots \\ {\Delta B}_{n-m+1}\end{array}\begin{array}{c}\begin{array}{c}{\Delta B}_{2}\\ {\Delta B}_{3}\\ {\Delta B}_{4}\end{array}\\ \vdots \\ {\Delta B}_{n-m+2}\end{array}\begin{array}{c}\begin{array}{c}{\Delta B}_{3}\\ {\Delta B}_{4}\\ {\Delta B}_{5}\end{array}\\ \vdots \\ {\Delta B}_{n-m+3}\end{array}\begin{array}{c}\begin{array}{c}\dots \\ \dots \\ \dots \end{array}\\ \ddots \\ \dots \end{array}\begin{array}{c}\begin{array}{c}{\Delta B}_{m}\\ {\Delta B}_{m+1}\\ {\Delta B}_{m+2}\end{array}\\ \vdots \\ {\Delta B}_{n}\end{array}\right]\left[\begin{array}{c}{\alpha }_{1}\\ {\alpha }_{2}\\ {\alpha }_{3}\\ \vdots \\ {\alpha }_{m}\end{array}\right]$$

where ${W}_{t}^{*}$ is the correction variable for each observation from $m$ to $n$ in $t$, $m$ is the maximum lag time, $n$ is the number of total observations in the dataset.

The elimination of the earth tide is the same as the elimination of the barometric effect, using least squares linear regression to find the unit response coefficient $\gamma$. After finding the value of $\gamma \left(\gamma \right)$, the stepwise earth tide response coefficient $A\left(\gamma \right)$ is calculated by summing over the stepwise response:

16 $$\mathrm{A}(\gamma )=\sum_{\gamma =1}^{\gamma }\gamma \left(\gamma \right)$$

Using the response function, calculate the earth tide correction for each observation:

17 $$\left[\begin{array}{c}{W}_{m}^{*}\\ {W}_{m+1}^{*}\\ {W}_{m+2}^{*}\\ \vdots \\ {W}_{n}^{*}\end{array}\right]=\left[\begin{array}{c}\begin{array}{c}{\Delta ET}_{1}\\ {\Delta ET}_{2}\\ {\Delta ET}_{3}\end{array}\\ \vdots \\ {\Delta ET}_{n-m+1}\end{array}\begin{array}{c}\begin{array}{c}{\Delta ET}_{2}\\ {\Delta ET}_{3}\\ {\Delta ET}_{4}\end{array}\\ \vdots \\ {\Delta ET}_{n-m+2}\end{array}\begin{array}{c}\begin{array}{c}{\Delta ET}_{3}\\ {\Delta ET}_{4}\\ {\Delta ET}_{5}\end{array}\\ \vdots \\ {\Delta ET}_{n-m+3}\end{array}\begin{array}{c}\begin{array}{c}\dots \\ \dots \\ \dots \end{array}\\ \ddots \\ \dots \end{array}\begin{array}{c}\begin{array}{c}{\Delta ET}_{m}\\ {\Delta ET}_{m+1}\\ {\Delta ET}_{m+2}\end{array}\\ \vdots \\ {\Delta ET}_{n}\end{array}\right]\left[\begin{array}{c}{\alpha }_{1}\\ {\alpha }_{2}\\ {\alpha }_{3}\\ \vdots \\ {\alpha }_{m}\end{array}\right]$$

The observation water level minus the atmospheric pressure correction value is the observation well water level after the influence of positive atmospheric pressure; the observation water level minus the earth tide correction value is the observation well water level after the correction of the influence of earth tide. The atmospheric pressure and earth tide factors are also taken into account to obtain the water level correction Eq. (18).

18 $$\Delta \mathrm{W}\left(\mathrm{t}\right)=\sum_{i=0}^{m}\alpha \left(i\right)\Delta \mathrm{B}\left(\mathrm{t}-\mathrm{i}\right)+\sum_{\gamma =0}^{n}\gamma \left(\gamma \right)\Delta \mathrm{ET}\left(\mathrm{t}-\upgamma \right)$$

Where $\gamma \left(\gamma \right)$ is the unit corresponding coefficient of the earth tide response, $\Delta \mathrm{ET}\left(\mathrm{t}-\upgamma \right)$ is the value of the change in earth tide at moment $\mathrm{t}-\upgamma$.

### Analysis of the main influencing factors of groundwater level

**Figure 6:**
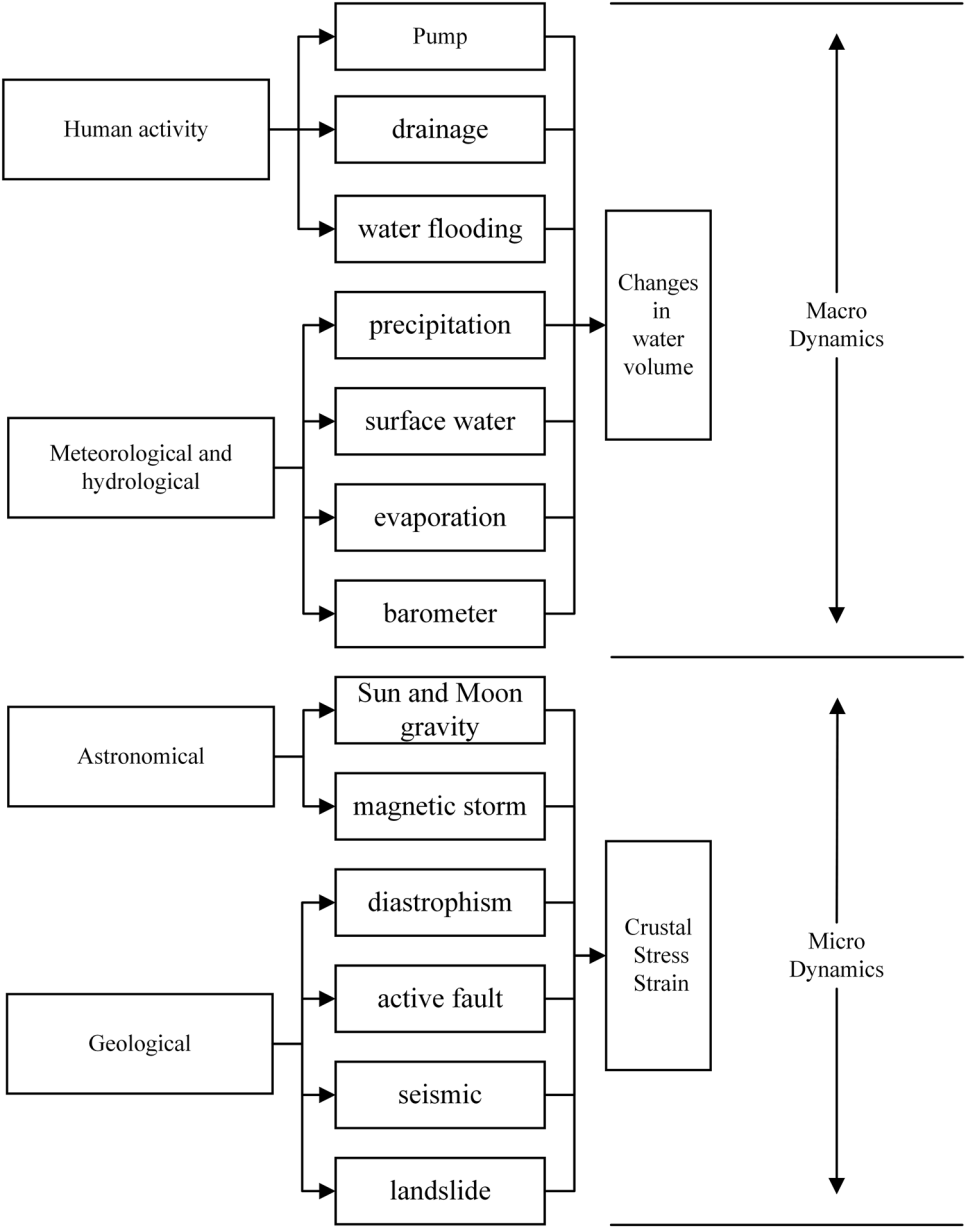
Analysis of the main influencing factors of groundwater level.

The variation of various elements of groundwater over time under natural and human factors is called groundwater level dynamics. Based on the formation mechanism of groundwater level dynamics, the influencing factors of groundwater level are divided into two categories: macroscopic and microscopic dynamics (Fig. 6): among them, the dynamic changes of water level caused by the increase or decrease of water in the aquifer are called macro dynamics, such as precipitation recharge, artificial mining, and water injection. For closed aquifers and semi-closed aquifers, these effects often have a long process, showing a trend change of half a month, a few months, or even a few years, which can be analyzed and calculated by the groundwater dynamics method. The stress change of the aquifer leads to the change of the pore water pressure of the aquifer, and the dynamic change of the water level caused by it is called microscopic dynamics. This variation puts emphasis on the dynamic properties and mechanics of the water table during stress–strain processes in aqueous media.

The monitoring well is located in the eastern mounding area of Fenggan Canal (name of the river) (Fig. 4b). affected by the landslide, the residents have moved to the west side of Fenggan Canal, there are no residents living on the slope, and after the site visit survey, there are no residents on the slope for pumping and draining activities in recent years. The nearest railroad is about 3.3 km away from the study area in a straight line, and according to the study of the ground vibration pattern around the area caused by the train operation^55^, the operation of the railroad will not affect the fluctuation of the water level of the well. The river level of Feng Gan Canal is stable all year round, and there is no significant rise or fall in water level, and the elevation of the river level is 398 m, and the elevation of monitoring wells is 528 m, with a relative height difference of 130 m and a distance of 760 m, therefore, the change of river level will not affect the change of well water level near the slip zone soil on the slope body; the formation thickness of the fourth system of monitoring wells in the area is about 8 m, and the area is a subtropical humid monsoon climate zone with abundant precipitation. There is no fault rupture in the area, so there is no geological condition for seismic activity, and there is no historical record of destructive earthquakes in the area, so there is no impact of earthquakes on groundwater during the monitoring measurement period. After the above analysis, the changes in the well water level in the study area are mainly influenced by atmospheric precipitation, barometer, earth tide, and landslides.

### Elimination of the main influencing factors of groundwater level

**Figure 7:**
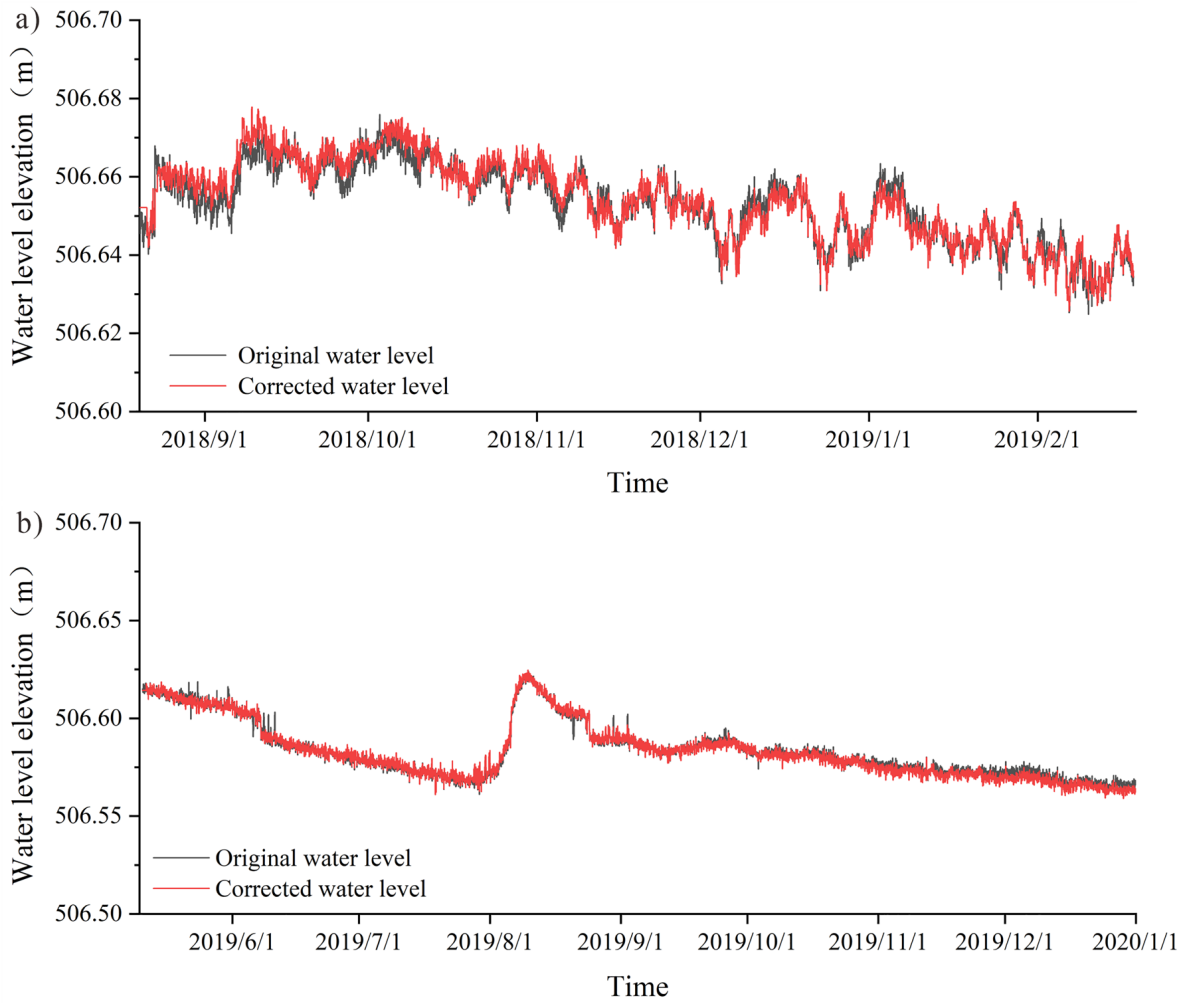
Water level change curve after eliminating barometer and earth tide.

**Figure 8:**
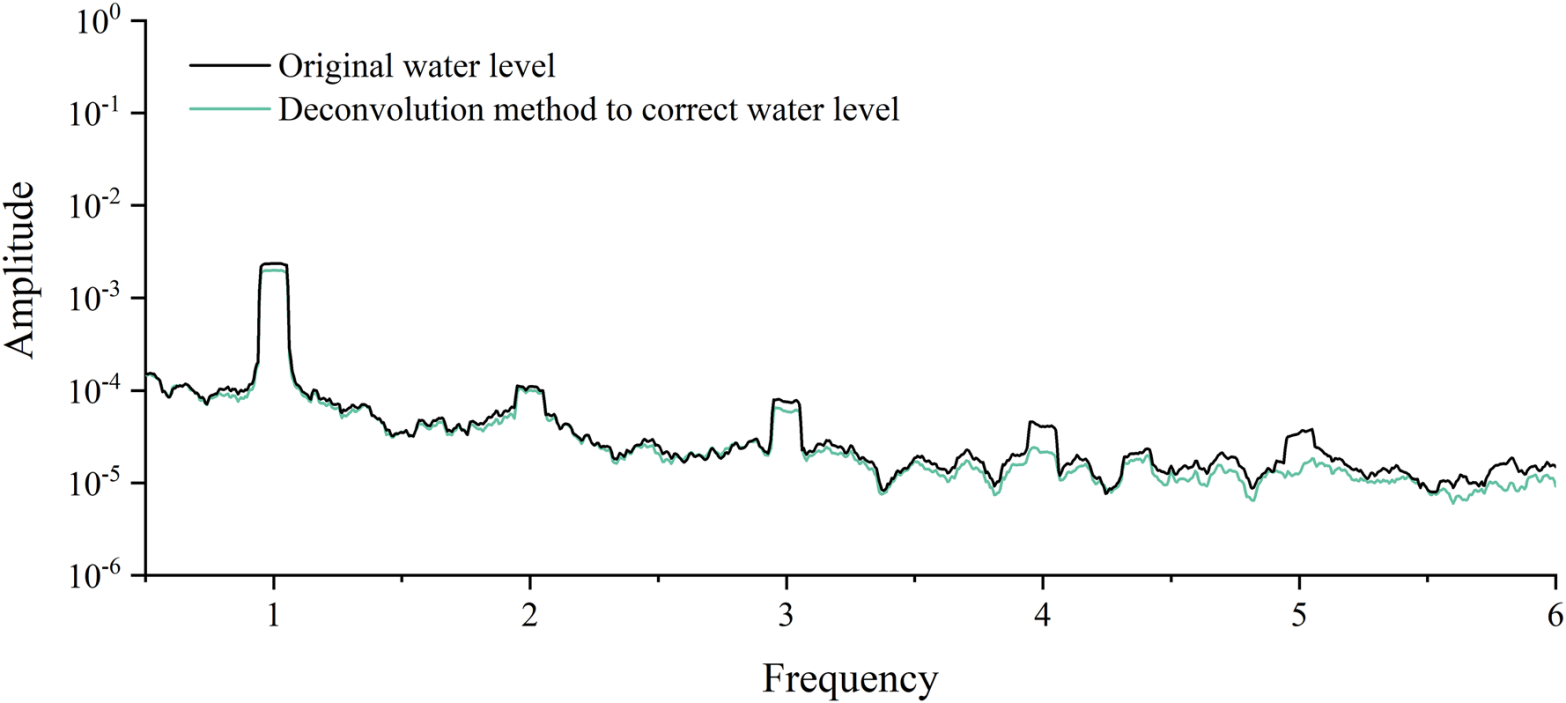
Water level spectrum after eliminating barometer and earth tide.

There are obvious daily, semi-diurnal waves, and 1/3 waves in barometer and earth tide. Although the nature of the forces and the mode of action of the earth tide effect and the barometric effect of the well water level are different, both tidal changes can cause tidal changes in the well water level^56^, while the action of barometer on water level has obvious hysteresis^57^. The inverse convolution regression method in the elimination of the barometer effect in the water level can consider the first dozen hours of barometer changes on the water level, so the barometer effect elimination using this method, using the same method to eliminate the earth tide effect in the water level, and finally get the water level change curve after the elimination of barometer, earth tide (Fig. 7), the water level time series graph of the elimination results through the Fourier transform for spectral analysis. The spectrogram shows (Fig. 8) that the water level peaks at 1 cpd, 2 cpd and 3 cpd have all been weakened, and the peak at 2 cpd (half-day cycle) has been weakened most obviously, indicating that the pressure on the water level, solid tide factors have been effectively eliminated.

**Figure 9:**
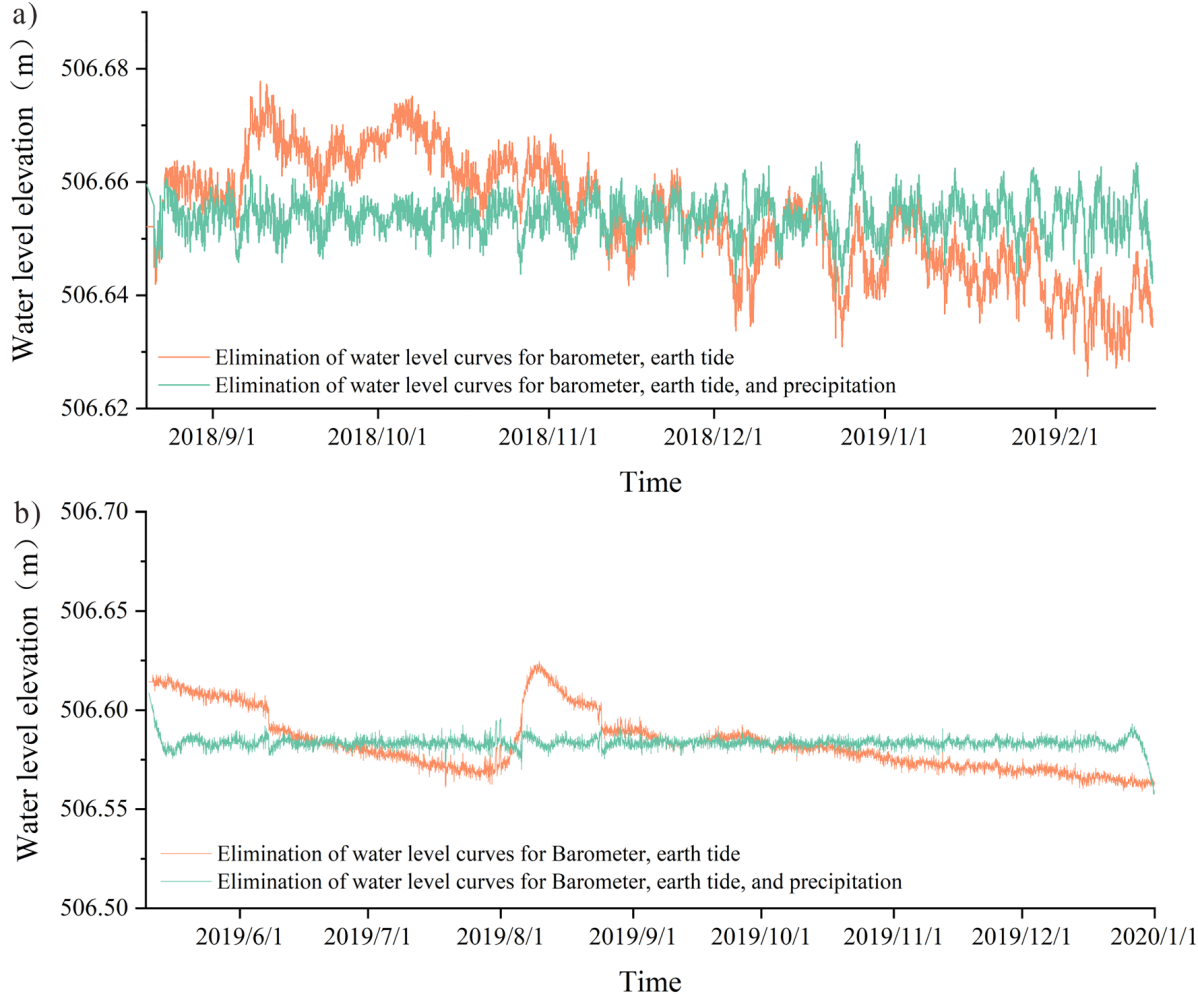
Graph of water level change after elimination of barometer, earth tide, and precipitation ((**a**) 2018.08.20–2019.02.18, (**b**) 2019.5.10–2019.12.31).

Through the statistical analysis of the precipitation frequency in the study area (Fig. 5 c), the precipitation frequency in the area is 0.1–0.5 cpd (circle per day, the number of daily cycles), for the groundwater level data after eliminating the influence of air pressure effect and solid tide effect, select the band-stop filter of 0.1–0.5 cpd to eliminate the influence of precipitation, and obtain the water level change curve after eliminating the influence of air pressure, solid tide and precipitation, as shown in Fig. 9, from the results, the groundwater level change after eliminating precipitation becomes more smooth and stable.

## Results and discussion

The 4 techniques are first evaluated on a synthetic dataset with known outliers. This is because the performance of different outlier detection algorithms cannot be evaluated if using real field data, since real production data is unlabeled and has no information about the type and number of outliers. Therefore, synthetic data with known outliers are used to quantitatively compare the performance of different algorithms. The hyperparameters of each technique are optimized to maximize its performance and select the best one. The selected techniques are then applied and evaluated on real field data..

### Evaluation of anomaly detection techniques on synthetic datasets

**Figure 10:**
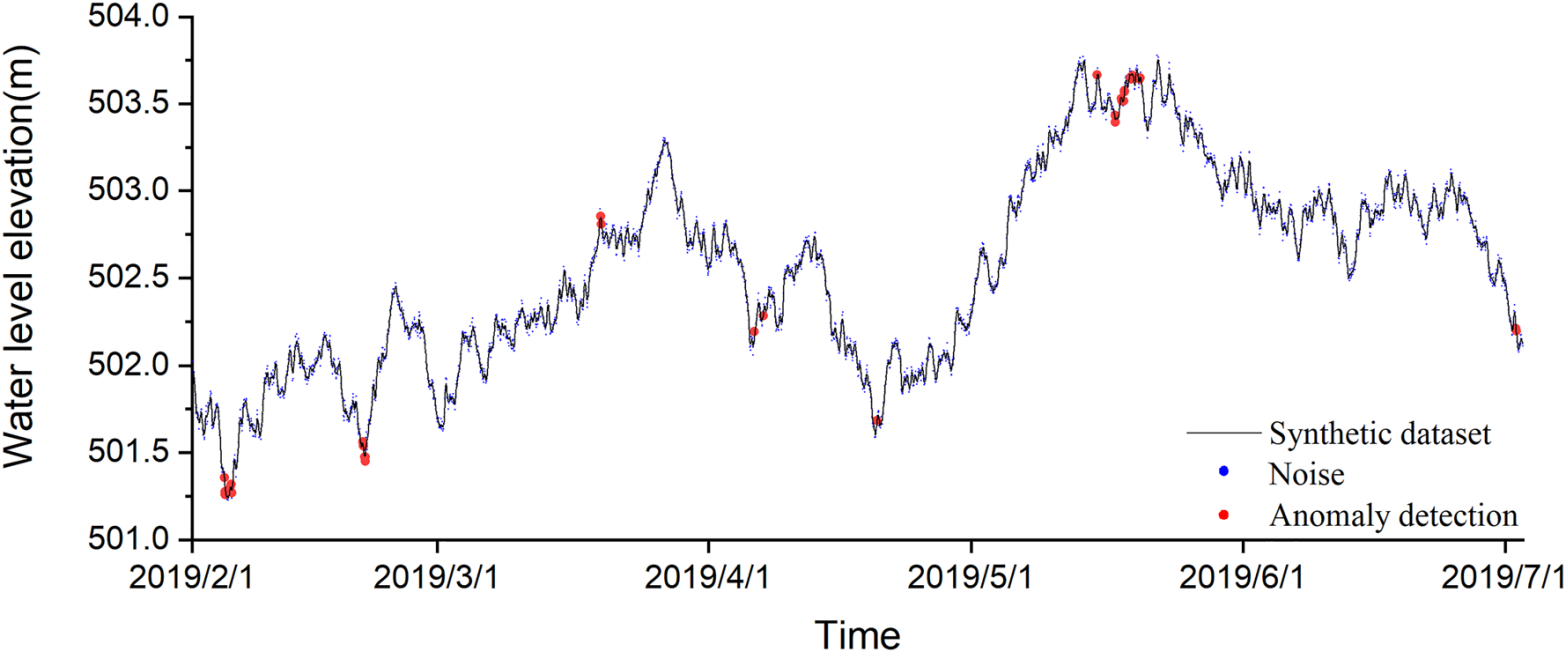
Synthetic data set, red dots are added outliers, and black lines are synthetic data.

Water level time series data from February to July 2019 were created using R software, the data volume is 3650, the frequency is 1 h, 1.5% white noise is added to the water level data, and the mutated points in the data were treated as outliers, a total of 35 outliers were filtered to constitute the final synthetic data set (Fig. 10). From Fig. 10, it can be seen that there are few outliers in the data and there is no clear boundary between normal and abnormal values, it is difficult to find the location of the outliers directly by visual inspection. Therefore, the above four machine learning techniques are used to detect outliers on synthetic data and evaluate the detection effect.

**Figure 11:**
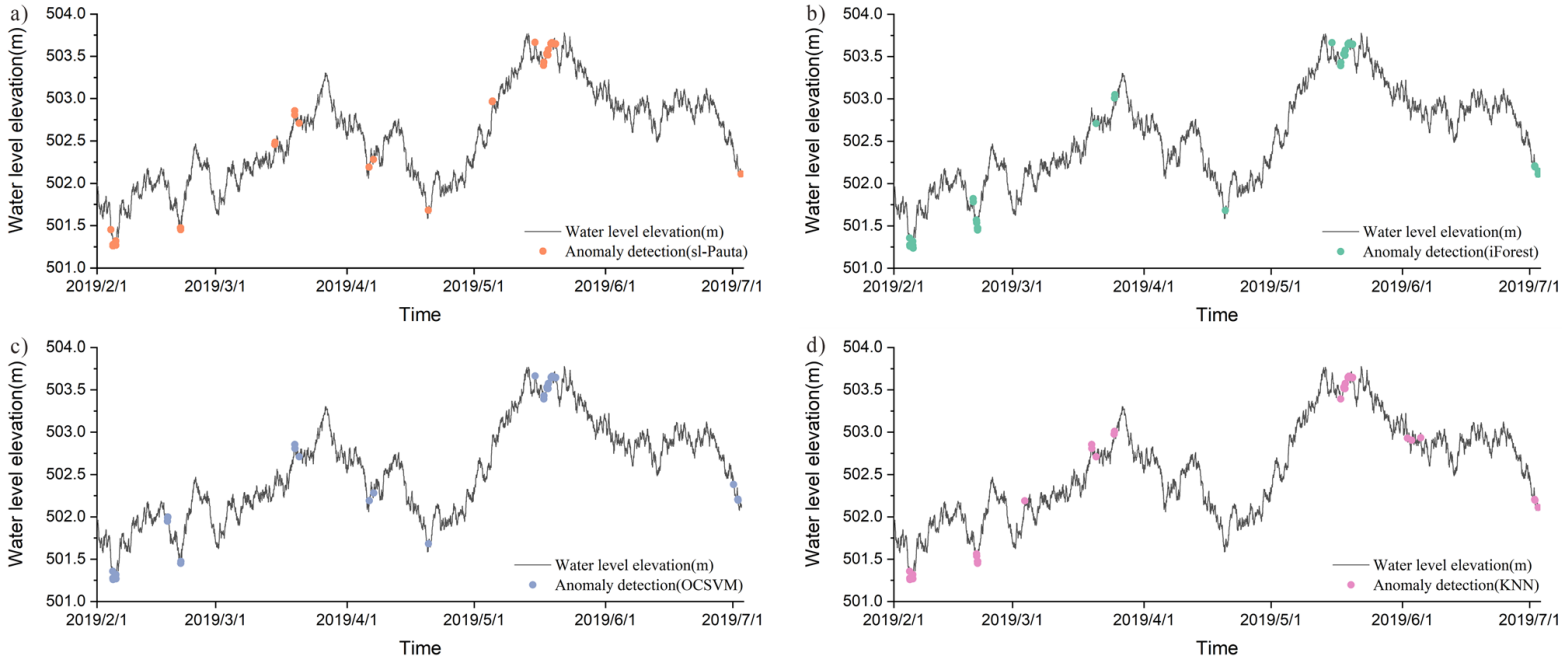
Outlier detection results for synthetic data.

**Figure 12:**
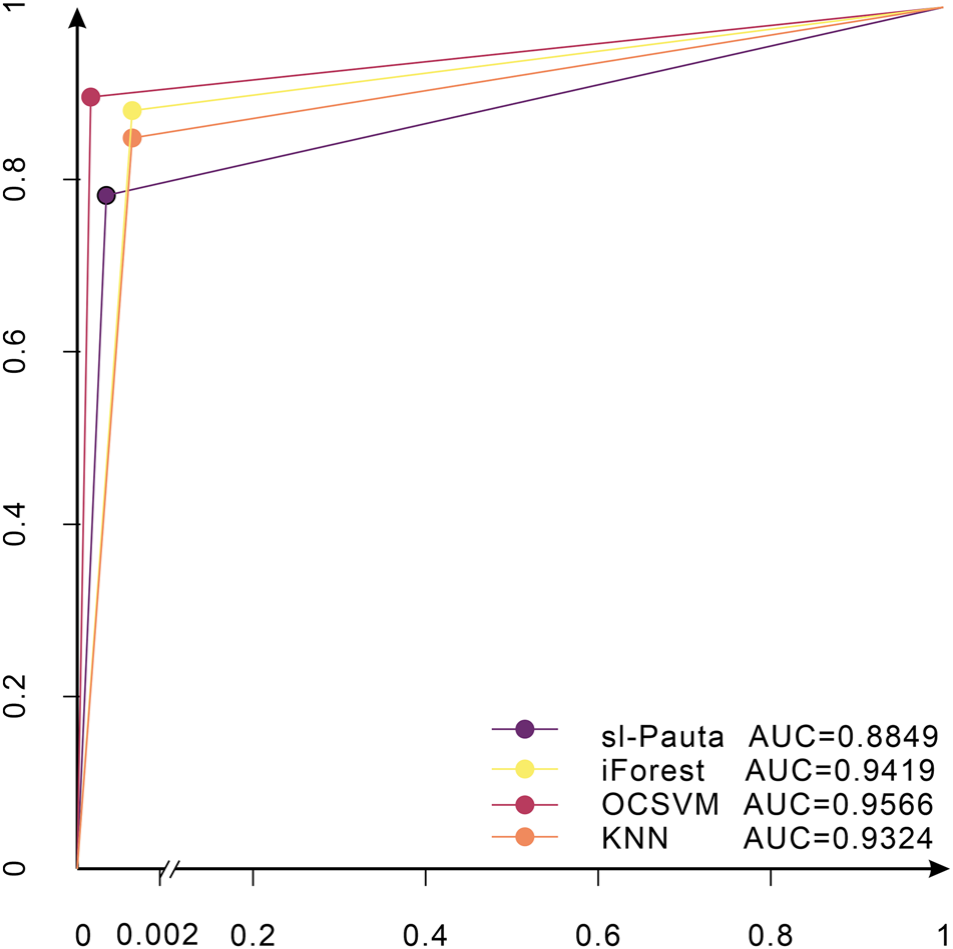
ROC curve evaluation results of the four algorithms.

It can be seen from the figure that all four methods detected the location of the anomalies to some extent (Fig. 11), but all four methods failed to mark all 35 anomalies accurately. The detection results of the four methods are given in Table 2. The sl-Pauta method detected 33 outlier results, of which 27 outliers were accurately identified, the number of normal values designated as outliers was 6, and 8 outliers were not detected. The iForest method detected 38 outlier results. Among them, 31 anomalies were accurately identified, the number of normal values designated as anomalies was 7, and the number of undetected anomalies was 4. The OCSVM method detected 36 anomalies, among which 32 anomalies were accurately identified, the number of normal values designated as anomalies was 4, and the number of undetected anomalies was 3. The KNN method detected 36 anomalies, among which 29 anomalies were accurately identified, the number of normal values designated as anomalies was 7, and the number of undetected anomalies was 6. In order to more intuitively evaluate the detection ability of the four methods, Precision, Recall, F1 score and AUC value were used as evaluation indexes (when the number of abnormal and normal values differed greatly, the evaluation effect of the accurary index would be greatly different in the case of unbalanced samples, so the index was not used in the article). The final evaluation results were calculated (Table 3), among which, the evaluation results of ROC curve method are shown in Fig. 12, and the results showed that all four methods performed well in terms of detection effect, and the OCSVM method had an precision rate of 88.89%, a recall rate of 91.43%, an F1 score of 90.14%, and an AUC value of 95.66%, which is the best detection effect; iForest and KNN methods have better detection effect than sl-Pauta, but the performance indexes of these two methods are low compared with OCSVM method.

**Table 2:** Test results of sl-Pauta, iForest, OCSVM, and KNN on the raw synthetic dataset.

	TP	FP	FN	TN
sl-Pauta	27	6	8	3609
iForest	31	7	4	3608
OCSVM	32	4	3	3611
KNN	29	7	6	3608

*TP* number of accurately detected abnormal values, *FP* number of normal values defined as abnormal, *FN* number of abnormal values defined as normal, *TN* number of normal values defined as normal.

**Table 3:** Performance comparison of sl-Pauta, iForest, OCSVM, and KNN on the raw synthetic dataset.

	Precision	Recall	F1 score	AUC
sl-Pauta	0.8182	0.7714	0.7941	0.8849
iForest	0.8158	0.8857	0.8493	0.9419
OCSVM	0.8889	0.9143	0.9014	0.9566
KNN	0.8478	0.8667	0.8571	0.9324

### Evaluation of anomaly detection techniques on processed datasets

Four detection methods in synthetic data are achieved a good detection effect, the four methods will be used for field real-time data detection for adaptability verification, because the field real-time monitoring data can not be marked in advance, and can not be used in common methods to evaluate the detection effect. Therefore, firstly, combined with the site, the site's real-time monitoring data were analyzed and factors eliminated to obtain simplified water level data (Fig. 9), then, the simplified water level data was imported into four methods for abnormal value detection, and the results were inserted into the original water level. The different algorithms were repeated to confirm whether they were truly abnormal values. In addition, the displacement data within the site was compared, and a qualitative analysis of the performance of the four methods was evaluated based on professional knowledge. The data were missing from February 19 to May 19, so the data were divided into two segments for testing from August 20, 2018, to February 18, 2019, and from May 10, 2019, to December 31, 2019.

**Figure 13:**
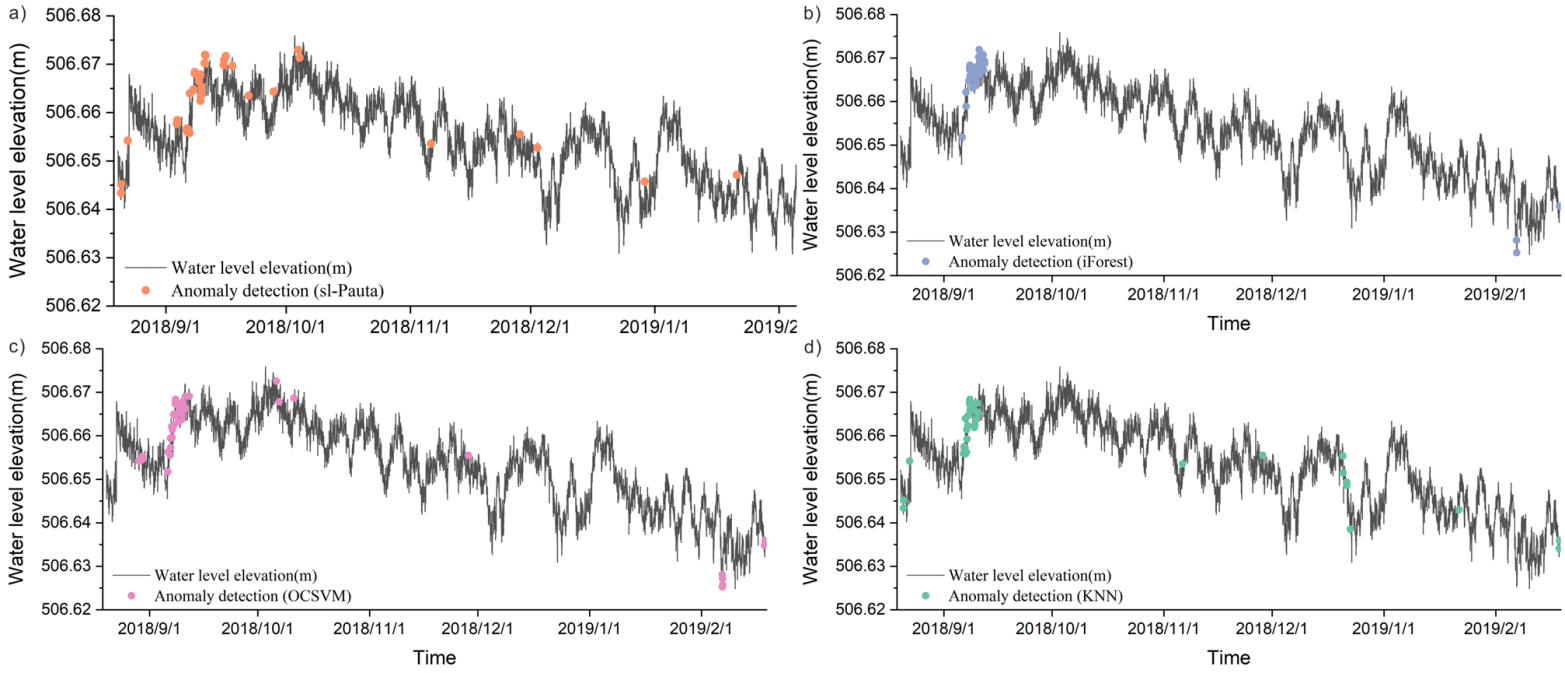
The plot of outlier detection results for actual data (2018.8–2019.2).

The detection results show that a total of 42 water level anomalies were identified by the sl-Pauta method during the time period of August 20, 2018 to February 18, 2019 (Fig. 13a), and the distribution of the anomalies on the time series shows that the anomalous values are mainly concentrated in the time period of 9.3–9.15 in 2018. A total of 44 anomalies were identified by the iForest algorithm (Fig. 13b), and the distribution of the anomalies on the time series shows that the anomalies are mainly concentrated in the time period of 9.7–9.12 in 2018. A total of 43 water level anomalies were identified by the OCSVM algorithm (Fig. 13c), and the distribution of the anomalies in the time series shows that the anomalous values are mainly concentrated in the time periods 9.6–9.10 in 2018 and 2019.2.6. A total of 43 water level anomalies were identified by the KNN algorithm (Fig. 13d), and the distribution of the anomalies in the time series shows that the anomalous values are mainly concentrated in the time period of 9.8.-9.10.2018. Through the visualization results of the data and the repeated testing results of four methods, it can be determined that the abnormal point main concentrated area in September 6–12, 2018.

**Figure 14:**
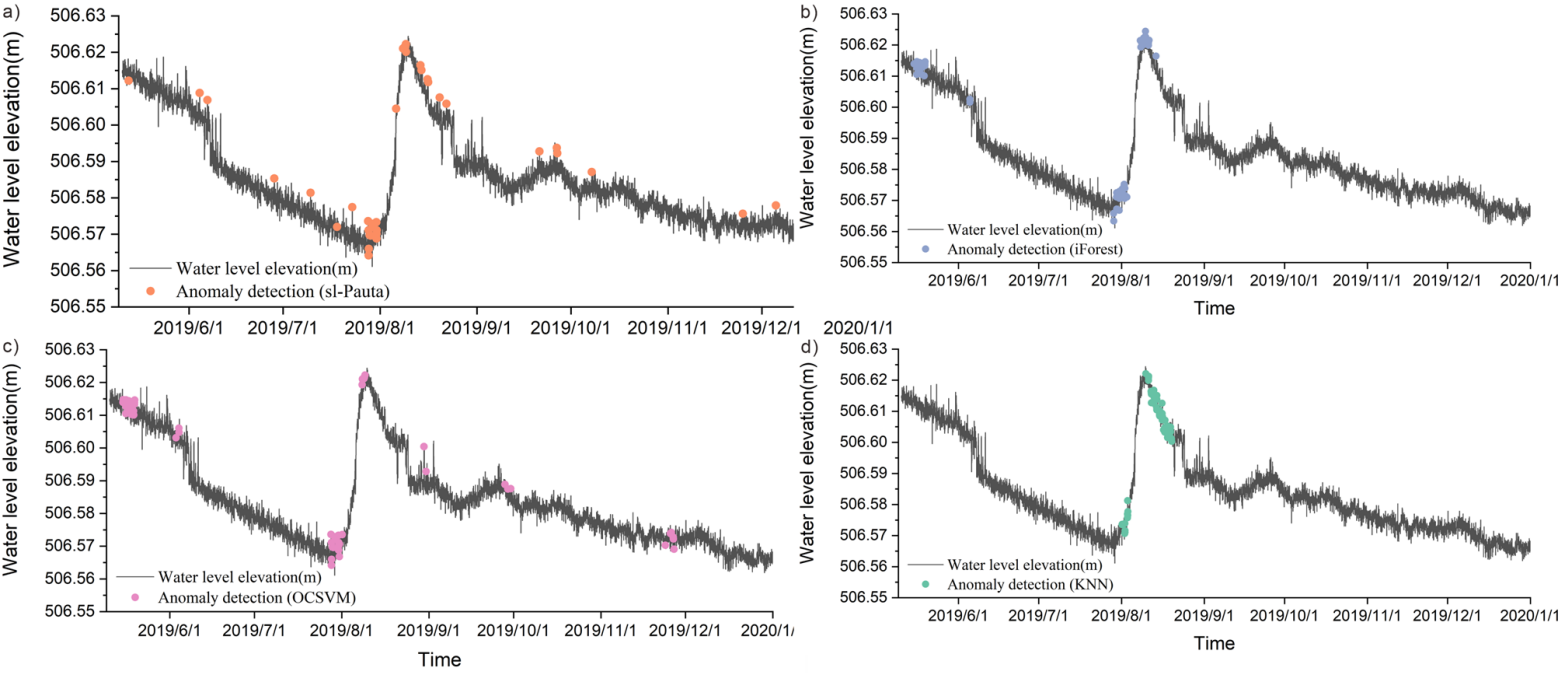
The plot of outlier detection results for actual data (2019.5–2019.12).

A total of 41 water level anomalies were identified by the sl-Pauta method in the time period of May 10, 2019–December 31, 2019 (Fig. 14a), and the distribution of the anomalies on the time series shows that the anomalies are mainly concentrated in the time period of 7.28–7.30 in 2019. A total of 57 water level anomalies were identified by the iForest algorithm (Fig. 14b), and the distribution of the anomalies on the time series shows that the anomalies are mainly concentrated in the time periods of 5.15–5.19, 7.29–8.3 and 8.8–8.11 in 2019. A total of 55 water level anomalies were identified by the OCSVM algorithm (Fig. 14c), and the distribution of the anomalies on the time series shows that the anomalous values are mainly concentrated in the three time periods of 5.15–5.19, 7.28–8.1, and 8.8–8.9 in 2019. A total of 56 water level anomalies were identified by the KNN algorithm (Fig. 14d), and the distribution of the anomalies on the time series shows that the anomalies are mainly concentrated in the 2 time periods of 8.1–8.3 and 8.11–8.19 in 2019. All four methods show that the anomalies are mainly concentrated in 7.28–8.3 of 2019, through the visualization results of the data and the repeated testing results of four methods, it was shown that the abnormal points were mainly concentrated in the period of July 28–August 3, 2019.

### Interpretation of anomaly detection

**Figure 15:**
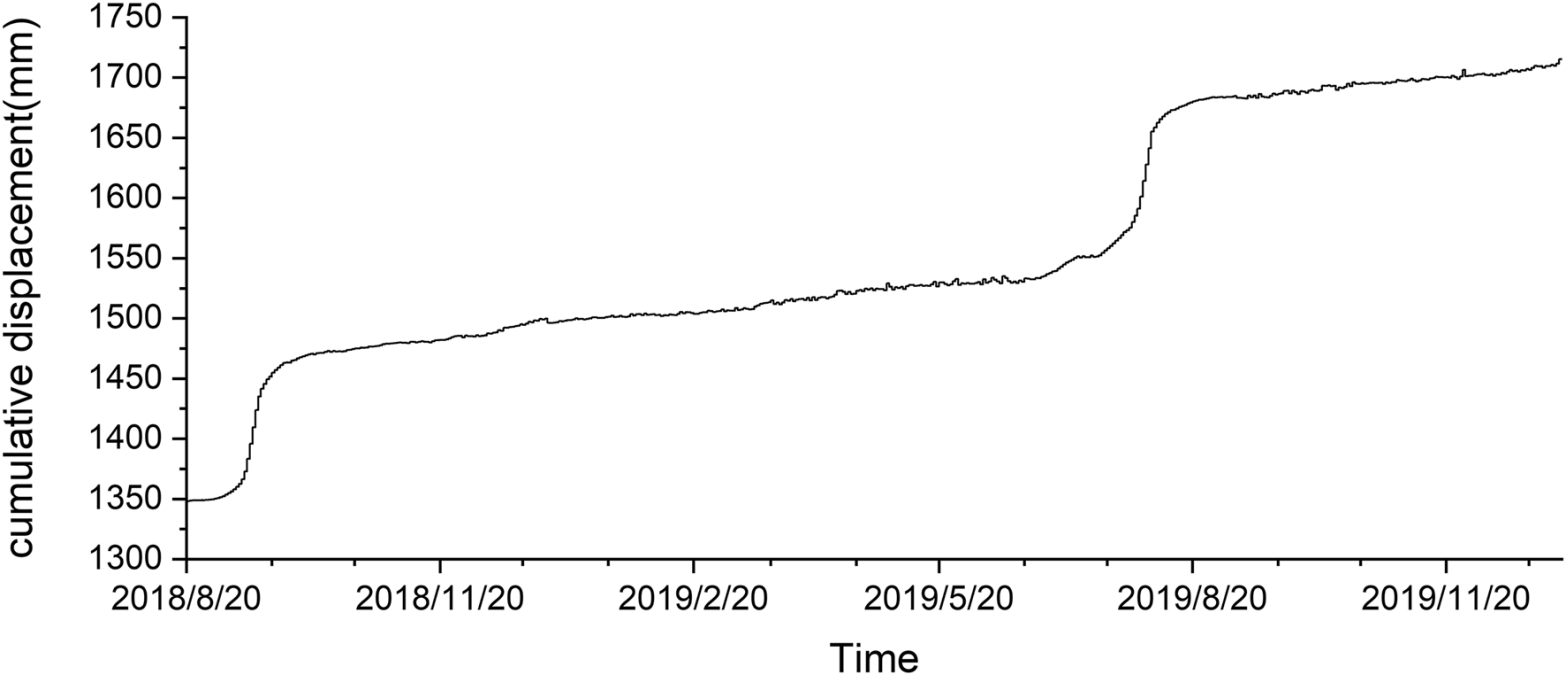
Cumulative displacement curve.

**Figure 16:**
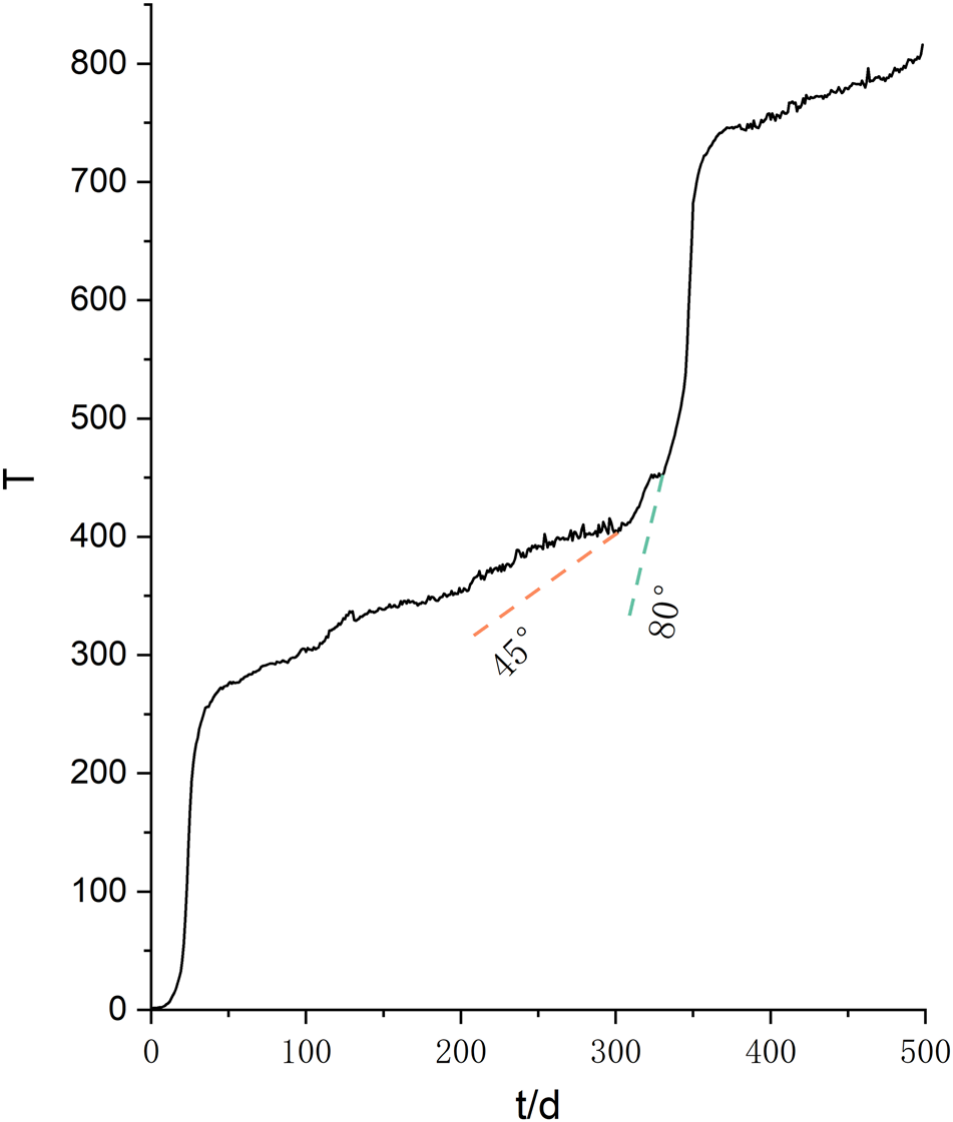
Displacement time series T–t curve.

Drawing the cumulative displacement–time curve of the landslide in the study area based on the characteristics that the displacement change law of different deformation stages of the slope will be reflected in the water level (Fig. 15)^58, 59^, and then test the outlier detection effect of the four methods by analyzing the relationship between displacement changes and outliers. When drawing the displacement–time curve of the slope, the dimensions used by different scholars are inconsistent, which leads to some defects in the displacement tangent angle method. In this paper, the improved tangent angle method proposed by XU Qiang's team^60^ was used to draw the T–t curve of the displacement time series during the research period (Fig. 16). The results of the T–t curve showed that there were two landslide events during the study period. The maximum tangent angle of landslide event a was 83°; the maximum tangent angle of landslide event b was 82°, and the landslides reached the alert level at 2:00 on September 8, 2018, and 2:00 on July 31, 2019, respectively. The mechanism of the role of groundwater in destabilizing landslides indicates that when the slope begins to slide, the stress on the rock and soil body gradually increases. The pores of the impermeable layer/weak pervious layer contain microcracks that close due to the compaction of the rock and soil body, resulting in a decrease in the porosity of the groundwater-bearing rock and soil body. As the porosity of the groundwater-bearing rock and soil body continues to decrease, the static water pressure within the system increases, causing water in the groundwater-bearing layer to exchange with the water in the wells only, resulting in an increase in the water level in the wells. After the slope has slid, the stress gradually decreases, and the static water pressure within the rock and soil body decreases. With the acceleration of the slope's slide, the static water pressure within the rock and soil body also decreases rapidly, allowing the groundwater-bearing layer to resume its permeability and restore the water-pressure exchange within the well-bearing system, resulting in a decrease in water level and a return to the state before the slide occurred^61, 62^. Comparing the detection results of water level anomalies, the water level detection results of the four methods all show that there are concentrated abnormalities in the water levels of 9.6–9.12 in 2018 and 7.28–8.3 in 2019. Therefore, it can be judged that the four methods have good effects in the outlier detection of water level time series. Further qualitatively comparing the characteristics of the outlier distribution in Figs. 13 and 14, iForest and OCSVM have better outlier detection, KNN has the second best detection effect, and the sl-Pauta has a relatively low detection effect.

**Figure 17:**
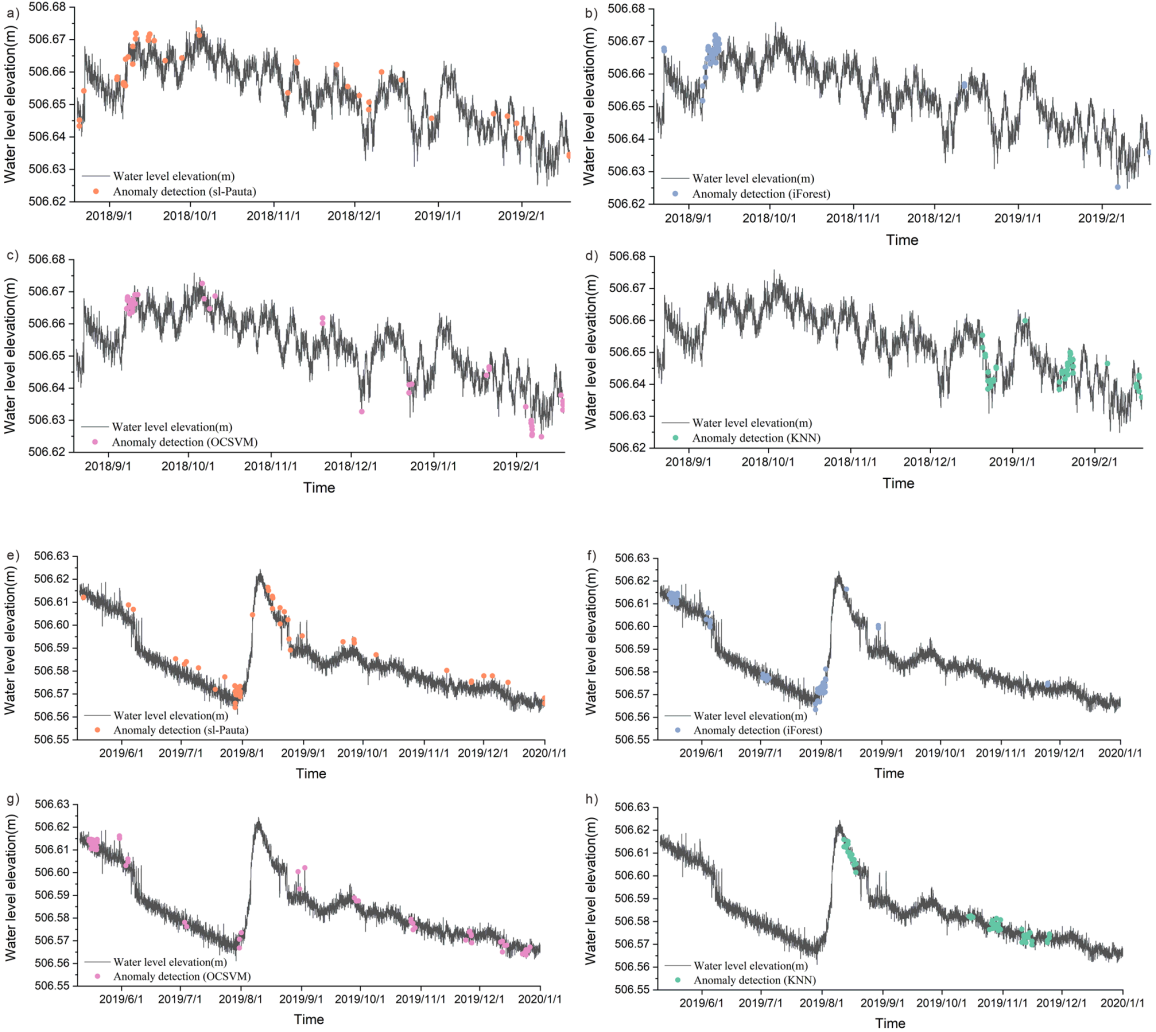
The plot of outlier detection results for raw data (**a**–**d** represents the detection results for the time period from August 2018 to February 2019, while **e**–**h** represents the detection results for the period from May 2019 to December 2019.)

Using four methods to detect abnormal water levels in raw data (Fig. 17), the detection results in both time periods were not satisfactory, mainly showing: during the same period, there was some overlap in the results of iForest and OCSVM, but there was no obvious clustering of abnormal values detected by the four methods overall. It is difficult to accurately determine abnormal data due to changes in external environmental conditions that can cause changes in data structure, which can affect the decision-making of algorithms. Compared to the results of simplified water level data detection (Figs. 13, 14) and the displacement T–t curve (Fig. 16), it can be seen that the groundwater detection method considering data structure has some advantages, providing a new idea for future groundwater abnormality detection.

## Conclusions

This article proposes a groundwater abnormal value detection method that considers the data structure, and implements and evaluates the performance of four machine learning algorithms. The results show that for the synthesized data set, OCSVM achieves the best detection performance, while iForest and KNN are good candidates for abnormal value detection. The detection technology ofsl-Pauta performs poorly. Secondly, the spectral analysis of water level shows that Kalman filter and inverse convolution regression can effectively remove the effects of rainfall, atmospheric pressure, and solid tide on water level, simplifying the factors affecting water level. Finally, the four technologies are used for simplified water level abnormal value detection, and the qualitative evaluation of detection effectiveness is conducted through the soil displacement–time curve of the site. The results show that the four methods have good effects in simplified water level abnormal value detection, with iForest and OCSVM achieving good abnormal value detection performance, KNN achieving intermediate performance, and sl-Pauta's detection performance being relatively low.

This study was primarily based on limited data and has not yet studied how this method can perform a series of abnormal value monitoring tasks on a wider range of data. Future work can collect more time series data from around the world to evaluate the advantages and disadvantages of this method against all available abnormal value detection algorithms currently available. However, the results of this study indicate that it may prove to be a valuable and useful supplement to existing abnormal value detection algorithms in this field.

### Supplementary Information

Supplementary Information.

## Data Availability

We declare that the Earth tide data used in this study was obtained from the Innovation Academy for Precision measurement Science and Technology, Chinese Academy of Sciences, while the remaining basic data was obtained through direct measurements taken by on-site monitoring instruments. Due to the need to retain the data for future research and to protect the privacy and security of the data, the generated and/or analyzed dataset during the current study is not publicly available. However, once the subsequent research is completed, the dataset will be made available at an appropriate time, and can be obtained upon reasonable request from the corresponding author Jian Guo, E-mail address: guojian2014@cdut.edu.cn.
