# Supplementary material for: Machine learning-based anomaly detection of groundwater microdynamics: case study of Chengdu, China
Source: Sci Rep. 2023 Sep 7;13:14718. doi: 10.1038/s41598-023-38447-5 (PMC10485069; doi:10.1038/s41598-023-38447-5)
Supplement: Supplementary file 1 — Supplementary Information. [file 41598_2023_38447_MOESM1_ESM.docx]

**Appendix 1.1.**

sl-Pauta algorithm

(1) Calculate the sample $X_{i} (i=1,2,3,\ldots\ldots,n)$ in the mean $\bar{x}$, $n$ being the number of samples;

(2) Calculate the standard deviation $\sigma$ based on the sample $X_{i}$ and the mean $\bar{x}$, $\sigma=\sqrt{\frac{\sum_{i=1}^{n} x_{i}-\bar{x}}{n-1}}$;

(3) Determine whether $x_{i+1},x_{i+2},x_{i+3},\ldots\ldots,x_{i+n}$ are abnormal by |x_(i+1)-x ̅ |>3σ. If the formula holds, then $x_{i+1}$ is an abnormal value, the abnormal value is marked and corrected by smoothing, otherwise, the value is normal;

(4) Let $x_{i}=x_{i+1}$, if $<n$, return to step (1) to repeat the execution, otherwise end the program.

When $x_{i+1}$ is an outlier, the correction equation is shown as

$$x_{i+1}=\frac{x_{i-2}+x_{i-1}+x_{i+1}+x_{i+2}}{4}$$
